# Can Economic Deprivation Protect Health? Paradoxical Multilevel Effects of Poverty on Hispanic Children’s Wheezing

**DOI:** 10.3390/ijerph110807856

**Published:** 2014-08-06

**Authors:** Timothy W. Collins, Young-an Kim, Sara E. Grineski, Stephanie Clark-Reyna

**Affiliations:** 1 Department of Sociology and Anthropology, University of Texas of El Paso, 500 West University Ave, El Paso, TX 79968, USA; E-Mails: segrineski@utep.edu (S.E.G.); seclarkreyna@miners.utep.edu (S.C.-R.); 2 Department of Criminology, Law and Society, University of California Irvine, 2340 Social Ecology II, Irvine, CA 92697, USA; E-Mail: youngank@uci.edu

**Keywords:** contextual effects, poverty, asthma, children, El Paso, Texas

## Abstract

Prior research suggests that economic deprivation has a generally negative influence on residents’ health. We employ hierarchical logistic regression modeling to test if economic deprivation presents respiratory health risks or benefits to Hispanic children living in the City of El Paso (Texas, USA) at neighborhood- and individual-levels, and whether individual-level health effects of economic deprivation vary based on neighborhood-level economic deprivation. Data come from the US Census Bureau and a population-based survey of El Paso schoolchildren. The dependent variable is children’s current wheezing, an established respiratory morbidity measure, which is appropriate for use with economically-deprived children with an increased likelihood of not receiving a doctor’s asthma diagnosis. Results reveal that economic deprivation (measured based on poverty status) at both neighborhood- and individual-levels is associated with reduced odds of wheezing for Hispanic children. A sensitivity analysis revealed similar significant effects of individual- and neighborhood-level poverty on the odds of doctor-diagnosed asthma. Neighborhood-level poverty did not significantly modify the observed association between individual-level poverty and Hispanic children’s wheezing; however, greater neighborhood poverty tends to be more protective for poor (as opposed to non-poor) Hispanic children. These findings support a novel, multilevel understanding of seemingly paradoxical effects of economic deprivation on Hispanic health.

## 1. Introduction

As the leading chronic disease during childhood in the industrialized world, asthma is a critical public health issue [1]. Research has revealed that asthma disparities are not attributable to one factor, but to multiple determinants that are interrelated and operate across individual and neighborhood levels. Understanding this complexity and successfully reducing asthma disparities requires use of a multilevel framework [2,3]. A multilevel approach can allow researchers to explain heterogeneities in asthma across socioeconomic and geographic boundaries that to date have remained largely unexplained. Most research has focused on individual-level risk factors and less attention has been given to the contexts in which people reside and the complex ways in which context may impact different types of people in different ways [2]. The advantage of a multilevel modeling approach, which we employ in this study, is that it enables examination of how contextual and individual factors may operate separately and in combination as influences on health outcomes.

This study examines economic deprivation as condition of material disadvantage and a hypothetical determinant of health outcomes. Based on prior research, we operationalize economic deprivation using measures of poverty status [4,5,6,7,8]. Most studies of the effects of neighborhood economic deprivation on respiratory health have not been multilevel. Ecological studies have tended to find neighborhood-level economic deprivation to be associated with higher aggregate asthma rates [9,10]. However, in an examination of El Paso County (Texas, USA)—which includes the study area for the analysis reported here—neighborhood poverty was not a significant predictor of children’s asthma hospitalization rates at the neighborhood level, and the coefficient was negative [11]. Despite several calls for adoption of a multilevel framework to better understand asthma disparities [2,3], only a few published studies have followed suit [1,12,13,14]. Recently, in Sweden, Li *et al*. [15] found that children were 8% more likely to be hospitalized for asthma in high deprivation as compared to low deprivation neighborhoods, controlling for maternal socio-demographics in a multilevel model. Neighborhood economic deprivation was associated with increased rates of asthma hospitalization independent of marital status, maternal educational attainment, urban/rural status, maternal history of asthma and smoking.

Neighborhood- and individual-level economic deprivation may have complex associations with children’s asthma. Some researchers have uncovered that while economic deprivation operated to increase risk of asthma at the individual level, higher socioeconomic status at the neighborhood level was a risk factor [1,14]. For example, using data from 20 large U.S. cities, Holt *et al*. [14] found that higher neighborhood education was associated with increased children’s asthma, even though the effect of mother’s education was the opposite at the individual level. In another study in Rhode Island, Pearlman *et al*. [16] found that economic well-being at the individual level (operationalized as having publicly-funded insurance) was a significant predictor of asthma while neighborhood economic deprivation (poverty) was only a significant predictor for non-Hispanic black children (as compared to their non-Hispanic white and Hispanic counterparts, even after controlling for other socioeconomic variables). Building off these studies, we examine the direct effects of neighborhood- and individual-level economic deprivation on children’s wheezing, but add the consideration of how neighborhood economic deprivation might impact the respiratory health of children differentially based on their individual-level deprivation status, which has not yet been investigated.

This study examines individual- and neighborhood-level determinants of current wheezing among Hispanic children living in El Paso, Texas using multilevel logistic regression modeling. The focus is on how economic deprivation at neighborhood- and individual-levels relates to children’s respiratory health and how the effects of neighborhood-level deprivation might vary based on children’s individual-level deprivation status (*i.e.*, for children within households in poverty *vs*. not in poverty). The twin foci of individual and neighborhood economic deprivation are important to consider together in Hispanic immigrant gateway contexts, such as El Paso, because Hispanic immigrants tend to be economically disadvantaged but in relatively good health. As an immigrant gateway city, El Paso provides a context in which to investigate the assumption that all deprived neighborhoods generate chronic stress that influences habitants’ health in negative ways. As Cagney *et al*. [17] note, some residential environments characterized as “deprived” may actually promote health.

The primary research questions and hypotheses are: (1) Does neighborhood economic deprivation significantly impact children’s current wheezing status independent of individual-level factors? Based on prior studies, we hypothesize that Hispanic children in neighborhoods with higher levels of economic deprivation will exhibit greater odds of wheezing, independent of individual-level economic deprivation. (2) Does individual-level economic deprivation significantly impact children’s current wheezing status independent of neighborhood-level economic deprivation? We hypothesize that Hispanic children with higher levels of individual/household economic deprivation will exhibit greater odds of wheezing, independent of neighborhood-level economic deprivation. (3) Does the effect of individual-level economic deprivation on Hispanic children’s wheezing vary based on neighborhood-level economic deprivation? There is not a solid basis for hypothesizing regarding research question 3, as this is a novel element of the analysis. However, we postulate that neighborhood economic deprivation will modify the association between individual-level poverty and wheezing, such that greater neighborhood economic deprivation will intensify the association between poverty and wheezing to a greater degree among poor children than it will among non-poor children.

## 2. Methods

### 2.1. Study Area

The study area is El Paso, Texas, which had an estimated population of 640,066 in 2011. In 2011, 80.7% of residents were Hispanic (compared to 16.1% of US residents and 37.2% of Texas residents). Smaller percentages of the city’s population were non-Hispanic white (14.7%) and non-Hispanic black (2.8%). According to recent US Bureau of the Census [18,19] figures, 23.3% of El Paso residents lived in poverty (compared to the US figure of 14.3%). Among El Paso residents, 25.5% were foreign-born (US = 12.8%) and 29.1% were less than 18 years of age (US = 24.0%). Additionally, El Paso has a children’s lifetime asthma prevalence rate of 17% [20]. This is relatively high compared to the Texas state children’s lifetime prevalence rate of 12% [21].

### 2.2. Study Design and Subjects

Individual-level data for El Paso children were collected through a population-based, cross-sectional, observational mail survey that was approved by our university’s Institutional Review Board. The closed-ended questionnaire was sent to all primary caretakers (parents and guardians) of fourth/fifth grade students in the El Paso Independent School District (EPISD). The survey was conducted using the tailored design method (TDM) to obtain the highest achievable response rates [22]. All survey materials were provided to households in English and Spanish. Mailings were sent in three waves during May of 2012. The first mailing consisted of the survey packet, which included a consent letter and the survey (in both English and Spanish), a $2 incentive and a postage-paid return envelope. A week later, we mailed a bilingual reminder postcard. One week after that, we re-sent the survey packet to all non-respondents (again with $2 and a postage-paid return envelope).

Ultimately, 6295 primary caretakers received surveys at their home address and 1904 were returned completed for a 30.2% response rate. Research indicates that similar and even substantially lower survey response rates can yield representative samples [23,24,25,26]. Respondents were primarily mothers (82%), fathers (10%) and grandparents (4%). More detailed descriptive statistics are presented below, but it is notable that the mean income of households was $20,000–$29,000. The vast majority of children (90%) were born in the US, and 16% were not continuously covered by health insurance over the past 12 months. This approximates the high rate of uninsured children in Texas, which leads the nation at 19% (the US average is 9%) [27]. Descriptive statistics indicate that the sample is generally representative [28]. The percent male and percent Hispanic are nearly identical between the sample and the EPISD as a whole (49.9% *vs*. 51.4% and 82.2% *vs*. 82.6% respectively); the sample has a lower percentage of economically-disadvantaged children than the EPISD (60.4% *vs*. 71.1%).

Based on reported home address in the survey, home locations for the 1901 individual children living in El Paso were geocoded. Because we examine neighborhood effects on health conditions occurring over the past 12 months, it was valid to include subjects only if children had lived at or near their current residence for 12 months or more. As an inclusion criterion, the survey question: “How long has this child lived within 1 mile (1.6 km) of your current residence?” was employed; the 337 participants responding “For less than 12 months” were excluded. We then excluded 204 non-Hispanic children from the analysis. Given racial/ethnic differences in asthma (e.g., in the US, blacks have substantially higher rates than Hispanics) and the low numbers of other races in the sample (e.g., only 112 children were black and 47 were Native American), we control for the effect of race/ethnicity by removing children of other racial/ethnic groups and focusing only on Hispanic children.

**Figure 1:**
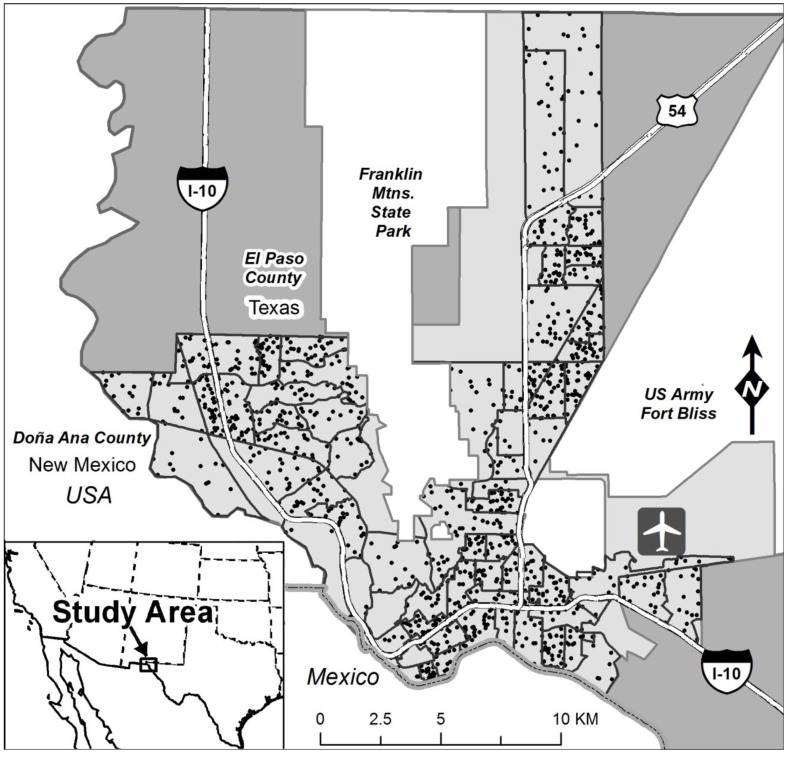
Approximate home locations of Hispanic children (black points) in census tracts (black bordered polygons) within the EPISD (light gray).

**Figure 2:**
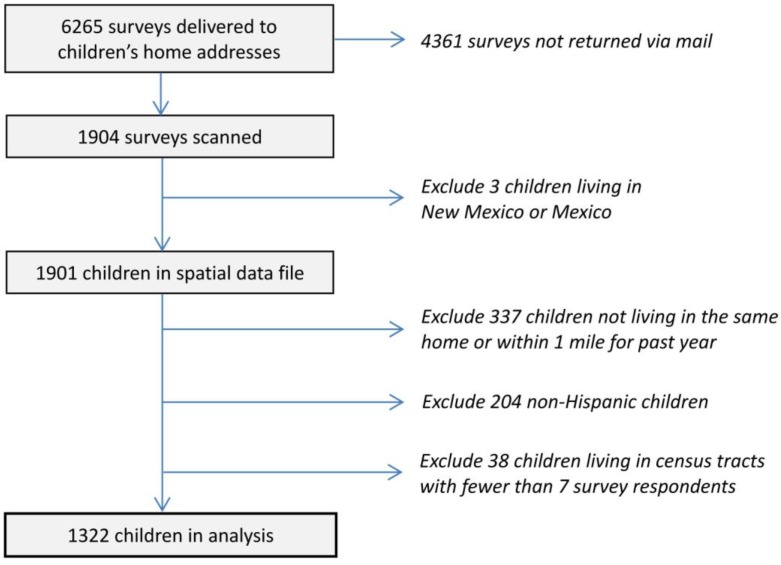
The EPISD survey sample.

The concept of a neighborhood is operationally defined as coinciding with 2010 census tract boundaries. This study includes each of 63 census tracts having at least seven Hispanic children, which we implemented as a cut-off for multilevel modeling. After excluding 38 remaining children located in census tracts containing less than seven participants, there were 1322 Hispanic children meeting all inclusion criteria. Figure 1 displays census tracts and approximate locations of children included in the analysis, while Figure 2 summarizes the process that yielded the survey data analyzed here.

Multiple imputation (MI) was applied to the individual-level dataset to address missing values and non-response bias, and the multiply imputed data were analyzed using HLM software. MI is currently a best practice for addressing missing data in statistical analysis. MI involves creating multiple sets of values for missing observations using a regression-based approach [29]. It is used to avoid the bias that can occur when missing values are not missing completely at random (MCAR) [29] and is appropriate for self-reported survey data [30]. In SPSS, 10 imputed datasets were specified to increase power and 200 between-imputation iterations were used to ensure that the resulting imputations were independent of each other. Using more than 3–5 datasets is the current “rule of thumb” in MI as it maximizes power and improves the validity of multi-parameter significance tests [30]. Analyzing a single imputed dataset treats the filled-in values as real data, which may underestimate sampling error. MI techniques appropriately adjust the standard errors for missing data [30]. HLM software allows for a maximum of 10 datasets in its MI option, and it accommodates the MI procedure by performing separate analyses on each dataset and then pooling results across analyses. The percent missing for the variables (described below) ranged from a low of <1% (general health status) to a high of 33% (obesity).

### 2.3. Dependent Variable

The outcome variable is children’s “current wheeze”, which has been used in prior studies [31,32,33,34,35]. Taken from the *International Study of Asthma and Allergies in Childhood*, the current wheeze item is a generally valid measure of asthma symptoms as responses to the question are in close agreement with asthma diagnosis by a respiratory physician [36]. The current wheeze variable is described in Table 1.

**Table 1:** Analysis variables: survey question, coding, and justification.

Variable	Survey Question	Coding	Justification
*Individual-level*			
Current Wheeze	Has your child had wheezing or whistling in the chest in the last12 months? From the International Study of Asthma and Allergies in Childhood (ISAAC)	1 = Yes 0 = No	Wheezing is a common symptom of asthma. It is easily recognized by parents and is more specific to asthma than cough [37]. Current wheeze was selected instead of diagnosed asthma due to this study’s emphasis on immigration, a characteristic that shapes access to medical care and thus an asthma diagnosis.
Sex (Male)	What is the child’s sex?	1 = Male 0 = Female	Boys have higher rates of asthma and other respiratory health problems than girls [38].
Age	What is the child’s age (in years)?	Continuous variable	Asthma rates vary by age [39].
Poverty Status	(a) How many people are living or staying at this address? (b) Which of the following best describes your yearly total household income for 2011 before taxes?	1 = Poor 0 = Non-poor	Asthma rates are higher for children of lower socioeconomic status [40,41].
General Health Status	How would you describe the overall health of the child? From the ISAAC	1 = Very good 0 = Not very good	Health status is used as a medical history variable to account for the child’s underlying state of health [42].
Obesity Status	(a) How tall is the child as of now? (b) How much does the child weigh as of now? (c) What is the child’s sex? (d) What is the child’s age (in years)?	1 = Obese 0 = Not obese	Being overweight is associated with higher rates of asthma generally [43], and among Hispanic children specifically [44].
Current Smoking	At any time during the past 12 months, has anybody smoked inside your child’s home?	1 = Yes 0 = No	Smoking inside homes is an important cause of respiratory illness [45,46].
Mold	Has your child’s home had mold or musty odors during the past 12 months?	1 = Yes 0 = No	Moldy/damp housing environments are associated with wheezing and asthma [47,48,49].
Nativity/Upbringing in El Paso (EP)	How long has this child lived in El Paso County?	1 = Born/raised in El Paso 0 = Not born/raisedin El Paso	Children’s asthma and allergy prevalence increased with longer residential duration in El Paso [50].
Primary Caretaker (PC) Born in the US	Primary caretakers were asked: “Where were you born?”	1 = US-born 0 = Foreign-born	Hispanic children born to US-born mothers have higher rates of asthma than those born to foreign-born mothers [51].
*Neighborhood-level*			
% Poverty (Economic Deprivation)	Percent of families residing in the census tract living below the poverty line	Continuous variable	Most but not all studies find neighborhood poverty to be a respiratory health risk factor [9,10,15].

### 2.4. Individual-Level Independent Variable

Individual-level co-variates—including child’s sex, child’s age, household poverty status, child’s general health status, child’s obesity status, smoking in the home, moldy odors in the home, child’s nativity/upbringing in El Paso, and primary caretaker’s nativity (US- or foreign-born)—are included as explanatory variables in this study because they have been associated with respiratory health outcomes in previous studies (see Table 1). Poverty and obesity status were constructed based on calculations employing multiple raw survey variables. Poverty status for each child’s household was calculated using caretaker-reported household size (a continuous variable for the number of people living in the home) and annual household income, based on guidelines for defining poverty status from the US Department of Health and Human Services [52]. Household income was reported by caretakers for 2011 on a 15-category scale, ranging from “Less than $1999” to “$150,000 or more”; mid-point values for caretaker-reported income categories were used in poverty status calculations. To determine obesity status, we first calculated body mass index (BMI) using caretaker-reported data on the child’s current height and weight; the child’s height was divided by the square of the child’s weight to derive BMI. We then used the age and sex of the child to determine if his/her BMI was above a 95th percentile threshold (*i.e.*, obese) or not, based on US Centers for Disease Control and Prevention guidelines [53]. The child’s general health status and nativity/upbringing in El Paso variables used in this analysis were recorded from ordinal scales into dichotomous indicators to address non-normality in the distributions of the original survey variables. General health status was reported by caretakers on a six-point Likert-type scale, ranging from “Very poor” to “Poor”, “Fair”, “Good”, “Very good” and “Excellent”. As per [54,55], we dichotomized the child’s general health status variable such that children in “Very good” or “Excellent” health were coded *1* and all other children were coded *0*. Child’s nativity/upbringing in El Paso was derived from a question asking how long the child had lived in El Paso, with five response categories ranging from “For less than 12 months” to “Since the child’s birth”. We dichotomized this variable such that children who were born and raised in El Paso were coded *1* and all other children were coded *0*.

### 2.5. Neighborhood-Level Independent Variable

Raw data to construct a variable for percent of families below the poverty line at the census tract level (*i.e.*, neighborhood economic deprivation) were downloaded from the US Bureau of the Census website [19] and transformed for analysis purposes (Table 1). We selected American Community Survey data for the years 2007–2011 [19] since they were the closest available match to our 2012 survey data.

### 2.6. Descriptive Statistics

Table 2 presents descriptive statistics for all individual- and neighborhood-level variables prior to multiple imputation. Approximately 14% of caretakers reported that their Hispanic children wheezed in the last 12 months. About 49% of the Hispanic children in this study are male. Approximately 43% of children live in households below the poverty line. Also, 8% and 13% of respondents reported smoking and moldy odors, respectively, in their homes in last 12 months. The mean value for percent of families in census tracts living below the poverty line is 23%. Ages of Hispanic children in the sample ranged from 7 to 13 years; 89% of children in this study were 9 to 11 years of age and 77% were 10–11 years of age.

**Table 2:** Descriptive statistics of analysis variables.

	N	Mean ^a^	SD	Min	Max	% Missing
*Individual*						
Current Wheeze	1268	0.14	0.35	0	1	2.42
Sex (Male)	1259	0.49	0.50	0	1	3.15
Age	1225	10.38	0.77	7	13	5.72
Poverty	1133	0.43	0.50	0	1	12.46
Excellent/Very Good Health Status	1291	0.70	0.46	0	1	0.59
Obesity	869	0.14	0.35	0	1	33.21
Smoking	1243	0.08	0.27	0	1	4.25
Mold	1247	0.13	0.33	0	1	4.03
Born/Raised EP ^b^	1148	0.69	0.46	0	1	11.44
PC ^c^ Born U.S.	1273	0.44	0.50	0	1	1.98
*Neighborhood*						
% Poverty	63	23.27	17.32	0.70	69.30	0

Notes: ^a^ Abbreviation for El Paso; ^b^ Abbreviation for Primary Caretaker; ^c^ The mean of a dichotomous variable (and all variables except for Age and % Poverty are dichotomous) can be interpreted as a proportion (e.g., the mean for current wheeze is 0.14, which means that 14% of the children had wheezed in the past year).

### 2.7. Analytic Strategy

Because this study is designed to assess the influence of neighborhood context on an individual-level binary dependent variable, we employed hierarchical logistic regression modeling (HLRM) to support accurate statistical inferences. HLRM enables multivariate analyses of multilevel data structures with binary outcomes. It is preferable to traditional logistic regression modeling for data analysis with different levels, because ignoring the hierarchical structure of data causes aggregation bias and leads to incorrect inferences [56].

Specifically, the level-1 equation analyzes the following variables: child’s sex, child’s age, poverty status, child’s health status, child’s obesity status, smoking behaviors, moldy odors, child’s nativity/upbringing in El Paso, and primary caretaker’s nativity. Percent poverty is included in the level-2 equation. First, determinants of current wheeze are modeled with only individual-level (level-1) variables (Model 1). Then, the neighborhood-level percent poverty variable is included in Model 2.

In order to address research question 3, it was necessary to analyze a cross-level interaction in addition to main effects. Model 3 includes an interaction between individual-level poverty status and percent poverty at the neighborhood level in addition to all main effects variables. Since HLM software does not perform multicollinearity diagnostic tests, SPSS was used to examine possible multicollinearity among the analysis variables included in each model. According to variance inflation factor, tolerance, and condition index criteria [57], inferences from the models do not appear to have been affected by multicollinearity problems. We also performed a sensitivity analysis of the reported findings to the choice of “current wheeze” as the dependent variable as compared to caretaker-reported “doctor-diagnosed asthma”.

## 3. Results

Table 3 displays the results for Models 1 and 2, which reveal determinants of Hispanic children’s wheezing status accounting for individual-level variables and percent poverty at the neighborhood level. Model 1 reports the effects of the individual-level variables on Hispanic children’s odds of current wheezing. Household poverty and having excellent/very good health status significantly predict reduced odds of wheezing. Child’s nativity/upbringing in El Paso and primary caretaker’s US-nativity are significantly and positively associated with current wheeze; *i.e.*, if a child was born/raised in El Paso or if their caretaker was US-born, the greater the likelihood that they had recently experienced wheezing. Moldy odor indoors was a risk factor that approaches significance in Model 1 (*p* < 0.10).

**Table 3:** Individual and neighborhood characteristics associated with children’s current wheeze.

Variables	Model 1			Model 2		
	Coef.	OR	95% CI	Coef.	OR	95% CI
*Intercept*	−0.628	0.534	(0.070, 4.051)	−0.466	0.627	(0.085, 4.617)
*Individual*						
Sex (Male)	0.236	1.266	(0.957, 1.675)	0.226	1.254	(0.948, 1.659)
Age	−0.146	0.864	(0.712, 1.049)	−0.135	0.874	(0.720, 1.061)
Poverty	−0.372 **	0.689	(0.505, 0.940)	−0.158	0.854	(0.591, 1.233)
Excellent/Very Good Health Status	−0.940 ***	0.391	(0.278, 0.548)	−1.000 ***	0.368	(0.258, 0.526)
Obesity	0.162	1.176	(0.762, 1.814)	0.204	1.226	(0.793, 1.895)
Smoking	−0.124	0.884	(0.478, 1.633)	−0.067	0.935	(0.509, 1.716)
Mold	0.408	1.503	(0.944, 2.394)	0.431	1.540	(0.965, 2.456)
Born/Raised EP^ a^	0.728 ***	2.072	(1.355, 3.167)	0.739 ***	2.094	(1.363, 3.218)
PC ^b^ Born US	0.734 ***	2.084	(1.547, 2.807)	0.692 ***	1.998	(1.485, 2.688)
*Neighborhood*						
% Poverty	--	--	--	−0.013 **	0.987	(0.978, 0.997)

Notes: *** *p* < 0.01; ** *p* < 0.05; ^a^ Abbreviation for El Paso; ^b^ Abbreviation for Primary Caretaker.

Model 2 includes percent in poverty (*i.e.*, economic deprivation) at the neighborhood level. Percent poverty significantly and negatively predicts Hispanic children’s wheezing (OR = 0.987, *p* = 0.011); *i.e.*, the higher the rate of poverty among families in the neighborhood, the lower the odds of wheezing among children (adjusting for individual-level poverty, along with all other individual-level variables). With the exception of individual-level poverty, which maintained its direction but became statistically non-significant (*p* = 0.224) in the presence of neighborhood percent poverty, all individual-level variables with statistical significance in Model 1 (*i.e.*, health status, nativity/upbringing in El Paso, primary caretaker born in US) show the same direction and significance in Model 2.

Table 4 reports the results of Model 3, which includes all level-1 variables, neighborhood-level percent poverty and a cross-level interaction between percent poverty at the neighborhood level and individual/household-level poverty status. In terms of individual-level main effects, all variables with significance in Model 2 (*i.e.*, health status, nativity/upbringing in El Paso, primary caretaker born in US) show the same direction and significance in Model 3. The neighborhood-level main effect of percent poverty also retains its directionality and significance. The cross-level interaction result reveals that the association of individual-level poverty with children’s wheezing was not statistically significantly modified by neighborhood percent poverty (economic deprivation). The direction of the relationship indicates that Hispanic children living in poverty (in comparison to those not in poverty) tend to exhibit even lower odds of wheezing as neighborhood economic deprivation increases (*p* = 0.345).

**Table 4:** Cross-level interaction, neighborhood percent poverty by individual poverty (Model 3).

Variables	Coef.	OR	95% CI
*Intercept*	−0.426	0.653	(0.088, 4.834)
*Individual*			
Sex (Male)	0.229	1.258	(0.950, 1.666)
Age	−0.131	0.877	(0.721, 1.067)
Poverty	−0.429	0.651	(0.365, 1.161)
Excellent/Very Good Health Status	−1.016 ***	0.362	(0.253, 0.519)
Obesity	0.203	1.225	(0.795, 1.888)
Smoking	−0.057	0.944	(0.516, 1.727)
Mold	0.430	1.537	(0.964, 2.449)
Born/Raised EP ^a^	0.747 ***	2.110	(1.380, 3.226)
PC ^b^ Born US	0.690 ***	1.994	(1.482, 2.683)
*Neighborhood*			
% Poverty	−0.017 **	0.983	(0.969, 0.998)
*Cross-level Interaction*			
% Poverty × Poverty (household)	0.008	1.009	(0.991, 1.027)

Notes: *** *p* < 0.01; ** *p* < 0.05; ^a^ Abbreviation for El Paso; ^b^ Abbreviation for Primary Caretaker.

In terms of model fit, Model 1 (deviance = 3548.1) has significantly better fit (*p* < 0.001) than the null model (deviance = 3627.2), and Model 2 (deviance = 3542.6) has significantly better fit (*p* = 0.018) than Model 1; while Model 3 (deviance = 3542.1) does not exhibit statistically significantly better fit (*p* = 0.194) than Model 1, we briefly present and discuss results due to their theoretical relevance. Results of the sensitivity analysis (tables not shown) reveal that the effects are generally the same for doctor-diagnosed asthma as they are for current wheeze. In Models 1 and 2, the statistically significant findings are the same as for current wheeze, except sex (male) becomes significant (*p* < 0.01), and, in Model 2, primary caretaker born in the US becomes statistically non-significant. Notably, in Model 1 for doctor-diagnosed asthma, individual poverty status significantly predicts lower odds of asthma diagnosis at the *p* < 0.05 level, and, in Model 2, neighborhood percent poverty significantly predicts reduced odds of asthma diagnosis at the *p* < 0.01 level (as is the case for current wheeze). In Model 3 for doctor-diagnosed asthma, the interaction term for neighborhood percent poverty × individual poverty has the same directionality as for current wheeze and is also statistically non-significant.

## 4. Discussion

This study examined multilevel effects of economic deprivation on Hispanic children’s wheezing and contributes to knowledge in a few regards. It provides a more nuanced analysis than prior studies by (a) examining contextual effects (research question 1), (b) compositional effects (research question 2) and (c) a cross-level effect (research question 3) of economic deprivation on respiratory health outcomes among Hispanic children residing in a Hispanic majority immigrant gateway located at the US-Mexican border (El Paso, Texas). By examining main effects at neighborhood- and individual-levels as well as a cross-level interaction effect, we can begin to disentangle the complex multilevel influences of economic deprivation on Hispanic children’s respiratory health.

In contrast to our hypothesis for research question 1, we found that neighborhood economic deprivation was negatively rather than positively associated with Hispanic children’s wheezing (*i.e.*, higher levels of poverty predicted lower odds of wheezing), accounting for individual-level economic deprivation (see Model 2). This is a counterintuitive contextual effect. A few published studies report a similar, inverse relationship between neighborhood economic deprivation and individual respiratory health problems [1,14,58]. Shankardass *et al*. [1] found that odds of individual diagnosed asthma decreased as community economic deprivation increased and that this association was stronger among Hispanics than it was among non-Hispanics. Based on this finding, Shankardass *et al*. [1] speculated that, in the context of economic deprivation, Hispanic communities may have collective advantages against asthma, possibly due to their enhanced social capital, compared with communities primarily of other racial/ethnic make-up.

Considering Shankardass and colleagues’ [1] results and the high correlations between neighborhood-level poverty, Hispanic and immigrant (foreign-born) concentration that exist in El Paso (based on census tract-level data, correlations not shown), it appears that the potentially detrimental respiratory health effects of economic deprivation may be attenuated by factors not measured via the survey, such as mutual support, collective efficacy, social ties, and exchanges of resources and information within poor Hispanic immigrant communities [17,59]. This suggests that the deleterious effects of economic deprivation on health outcomes may be collectively mediated by strong social and cultural ties that tend to be constituted within certain racial/ethnic minority communities.

In contrast to our hypothesis for research question 2, we found individual-level economic deprivation to be negatively rather than positively associated with children’s wheezing. To our knowledge, few prior studies have found individual-level poverty to be significantly protective of respiratory health. The negative directional relationship held across Models 1–3, and it was significant in Model 1. However, the statistical significance of the protective individual-level effect of poverty (in Model 1) did not persist when accounting for neighborhood-level poverty in Model 2. This indicates that the protective compositional (individual) effects of household poverty on Hispanic children’s respiratory health in El Paso are explained in part by the protective contextual (neighborhood) effects of concentrated economic deprivation.

In terms of research question 3, the cross-level interaction result (from Model 3) enables us to address Wright and Subramanian’s [2] call to investigate how neighborhood characteristics impact different types of people in divergent ways. Our results indicate that the protective effect of individual-level poverty is not statistically significantly modified by living in economically-deprived contexts; however, based on the directionality of the relationship, greater neighborhood economic deprivation tends to be more protective for Hispanic children in poverty (*vs*. not in poverty). The finding that greater neighborhood economic deprivation is associated with even less wheezing for Hispanic children in poverty (*vs*. not in poverty) runs counter to our hypothesis. Rather than working synergistically to the detriment of children’s respiratory health, neighborhood- and individual level economic deprivation may interact in a protective manner, which might be explained by the greater access to mutual support enjoyed among poor Hispanic people within El Paso’s generally economically-deprived Hispanic neighborhoods. The direction of the interaction effect relates to the surprising main effect of individual-level poverty across the models. Few prior studies have found economic deprivation to be protective of respiratory health at the individual level. Together, these findings lend support to the thesis that strong social and cultural ties within poor Hispanic communities may serve to attenuate the detrimental effects of economic deprivation on health [60]. In other words, as Wen *et al*. [61] suggest, neighborhood economic deprivation might work through social resources to influence health status.

## 5. Conclusions

In sum, a multilevel analysis approach enabled examination of contextual, compositional and cross-level effects of economic deprivation on health. This allowed for the documentation of paradoxical effects, in which economic deprivation was apparently beneficial for Hispanic children’s respiratory health. Interestingly, a recent county-level study of Texas Hispanic adults found a similarly paradoxical pattern: While greater economic deprivation was associated with higher mortality of several cancers among non-Hispanic whites and blacks, it was associated with *lower* mortality among Hispanics [62]. The authors of that study speculated that this anomaly might be primarily attributable to dynamics occurring within Texas’ Mexican border counties (which include El Paso County). We argue that such paradoxical results—wherein economic deprivation is protective of health—might not be found in most other contexts, since stable, yet impoverished racial/ethnic minority communities characterized by strong social ties are not common in the US. Several features of El Paso’s context, which were not accounted for in our models, might be influencing the reported findings. While El Paso is among the poorest of US cities (with a Hispanic majority), it paradoxically has among the lowest violent crime rates in the nation [63]. In other cities, concentrated economic deprivation is commonly associated with increased violence, which has been linked to stress and high asthma rates [2]. There are also high levels of home ownership and residential stability in El Paso’s poor neighborhoods. Additionally, El Paso’s vast Hispanic majority context may provide collective advantages to Hispanic children, in spite of their generally relatively high levels of economic deprivation.

Finally, it is important to note that very few Hispanic children in our sample experience absolute economic deprivation. These children are relatively disadvantaged economically within a wealthy country that offers some semblance of a social safety net. The majority of impoverished Hispanic children in our sample are US citizens covered by government-subsidized healthcare. We hypothesize that, in other cases wherein very few individuals experience absolute deprivation and in which many economically-disadvantaged individuals receive healthcare entitlements, relationships between economic deprivation and health outcomes are likely to be complex and modified by social and environmental factors. More longitudinal and multilevel research is needed to foster a multidimensional understanding of protective and risk factors. In future research, longitudinal study designs should be employed in order to disentangle complex causal pathways, which could not be fully clarified based on the cross-sectional study design employed here. Future multilevel modeling analyses should include neighborhood-level measures of collective efficacy or social capital (e.g., [17]) and exposure to environmental triggers (air pollution for respiratory health) alongside economic deprivation to explore these relationships across a range of social contexts.
